# Effects of Kifunensine on Production and *N*-Glycosylation Modification of Butyrylcholinesterase in a Transgenic Rice Cell Culture Bioreactor

**DOI:** 10.3390/ijms21186896

**Published:** 2020-09-20

**Authors:** Kantharakorn Macharoen, Qiongyu Li, Veronica A. Márquez-Escobar, Jasmine M. Corbin, Carlito B. Lebrilla, Somen Nandi, Karen A. McDonald

**Affiliations:** 1 Department of Chemical Engineering, University of California, Davis, CA 95616, USA; kmacharoen@ucdavis.edu (K.M.); vero_marquez_333@hotmail.com (V.A.M.-E.); jasminemcorbin@gmail.com (J.M.C.); snandi@ucdavis.edu (S.N.); 2 Department of Chemistry, University of California, Davis, CA 95616, USA; qyuli@ucdavis.edu (Q.L.); cblebrilla@ucdavis.edu (C.B.L.); 3 Facultad de Ciencias Químicas, Universidad Autónoma de San Luis Potosí, San Luis Potosí 78210, Mexico; 4 Sección de Biotecnología, Centro de Investigación en Ciencias de la Salud y Biomedicina, Universidad Autónoma de San Luis Potosí, San Luis Potosí 78210, Mexico; 5 Global HealthShare® Initiative, University of California, Davis, CA 95616, USA

**Keywords:** butyrylcholinesterase, plant cell suspension cultures, kifunensine, *N*-glycosylation, plant-made biopharmaceuticals

## Abstract

The production and *N*-glycosylation of recombinant human butyrylcholinesterase (BChE), a model highly glycosylated therapeutic protein, in a transgenic rice cell suspension culture treated with kifunensine, a strong α-mannosidase I inhibitor, was studied in a 5 L bioreactor. A media exchange was performed at day 7 of cultivation by removing spent sugar-rich medium (NB+S) and adding fresh sugar-free (NB-S) medium to induce the rice α-amylase 3D (RAmy3D) promoter to produce rice recombinant human BChE (rrBChE). Using a 1.25X-concentrated sugar-free medium together with an 80% reduced working volume during the media exchange led to a total active rrBChE production level of 79 ± 2 µg (g FW)^−1^ or 7.5 ± 0.4 mg L^−1^ in the presence of kifunensine, which was 1.5-times higher than our previous bioreactor runs using normal sugar-free (NB-S) media with no kifunensine treatment. Importantly, the amount of secreted active rrBChE in culture medium was enhanced in the presence of kifunensine, comprising 44% of the total active rrBChE at day 5 following induction. Coomassie-stained SDS-PAGE gel and Western blot analyses revealed different electrophoretic migration of purified rrBChE bands with and without kifunensine treatment, which was attributed to different *N*-glycoforms. *N*-Glycosylation analysis showed substantially increased oligomannose glycans (Man5/6/7/8) in rrBChE treated with kifunensine compared to controls. However, the mass-transfer limitation of kifunensine was likely the major reason for incomplete inhibition of α-mannosidase I in this bioreactor study.

## 1. Introduction

Protein expression in plant systems has the potential to provide a safe, cost-effective, and scalable method to meet the increasing need for therapeutic protein production. Plant-based expression offers several advantages to the biopharmaceutical industry, including decreased cost of production, scalability, lack of susceptibility to mammalian pathogens, elimination of animal- or human-sourced raw materials, and the production of complex proteins with post-translational modifications such as *N*-glycosylation [1,2,3,4,5]. For many therapeutic proteins, *N*-glycosylation is essential for protein folding, oligomerization, quality control, enzyme activity, ligand interactions, localization, and trafficking [6,7].

Despite its potential, a possible barrier to the commercialization of plant-made glycoprotein drugs is the difference between the *N*-glycan structures of human and plants. Of particular concern are plant-specific structures contained in complex type *N*-glycans, namely, α1,3 core fucose, β1,2 bisecting xylose, and the Lewis a epitope [8]. Even though there is no definitive proof of adverse effects from plant-specific glycan structures [9], the presence of nonhuman glycans could potentially cause unwanted immunogenicity in humans, and the lack of sialic acid termination may lead to reduced blood circulatory half-life. A change in glycan structure could also potentially alter the protein’s structure or accessibility of its epitopes and, consequently, its function. Therefore, to ensure the efficacy of a plant-made biosimilar therapeutic, it is important that the *N*-glycans are compatible with both the protein’s function and the human immune system.

Several strategies exist to modify a glycoprotein’s *N*-glycan structures in planta, such as glycoengineering of the host cells using CRISPR/Cas9 genome editing to knock out β(1,2)-xylosyltransferase (*XylT*) genes and α(1,3)-fucosyltransferase (*FucT*) genes [10,11,12] and RNA interference (RNAi) technology to downregulate *XylT* and *FucT* genes [13,14,15,16,17], targeting of the protein to specific organelles [18,19,20,21], addition of compounds to alter the function of glycan-modifying enzymes [4,22,23,24,25,26], and in vitro glycan remodeling using chemoenzymatic reactions [27]. In this work, we utilize kifunensine, a potent and highly specific inhibitor of α-mannosidase I in both plant and animal cells resulting in production of glycoproteins containing predominantly Man_8_GlcNAc_2_ (Man8) and Man_9_GlcNAc_2_ (Man9) structures [28], to a rice cell suspension culture grown in a bioreactor to inhibit α-mannosidase I activity. More than a few studies of kifunensine treatment in whole *Nicotiana benthamiana* plants successfully produced predominant Man9 structure glycoproteins [4,22,23,24,26]; however, the study of kifunensine treatment in plant cell suspension cultures, including transgenic rice cell suspensions [25], is very limited. In transgenic rice cell suspensions treated with 5 μM kifunensine cultivated in shake flasks, the productivity of a target glycoprotein, acid α-glucosidase (GAA), was significantly lower than the control, but the relative abundance of high-mannose structure GAA increased by 65% compared to the control [25]. Here, we report the effects of kifunensine treatment on production and *N*-glycosylation of a glycoprotein conducted in a bioreactor. The culture media, method of cultivation, degree of glycosylation, and multimerization of the product were different from the study by Choi et al. [25].

Rice (*Oryza sativa*) is generally recognized as safe (GRAS) by the FDA [29], and the rice alpha amylase 3D (RAmy3D) promoter, a metabolically-regulated strong promoter, used in this study was well studied [30,31,32,33,34,35,36,37]. In addition, semicontinuous bioreactor operations of transgenic rice cell suspensions proved the stability and robustness of transgenic rice cells under the RAmy3D promoter system for long-term recombinant protein production [38,39,40]. In this study, we use a transgenic rice cell suspension culture to produce recombinant human butyrylcholinesterase (BChE), a bioscavenger hydrolase enzyme that can be used as a therapeutic and prophylactic treatment to counter organophosphorus nerve agents [41], as a model glycoprotein. Human BChE is a tetrameric glycoprotein with four identical 69-kDa (not including oligosaccharides) monomers containing nine *N*-glycosylation sites per each monomer, with its activity, stability, and blood circulatory half-life highly dependent on the presence and structure of these glycans [41]. The production of recombinant human BChE in transgenic rice cell suspension cultures (rice recombinant BChE (rrBChE)) is controlled by the RAmy3D promoter that is highly activated in the absence of sugar [35,36,37,38,39,40,42].

Like other glycoproteins, *N*-glycosylation of a nascent rrBChE starts in the ER by co- or post-translational transfer of Glc_3_Man_9_GlcNAc_2_ from a dolichol lipid carrier onto Asn-X-Ser/Thr residues, where X is any amino acid except Pro [8]. Since our rrBChE gene construct contains the RAmy3D signal peptide, a glycosylated rrBChE follows the secretory pathway for secretion, involving removal of glucose and mannose residues and addition of new sugar residues (e.g., fucose, xylose, or galactose) and *N*-acetylglucosamine (GlcNAc), leading to complex-type *N*-glycans of rrBChE, as previously reported [3]. Our goal in this study is to investigate the effects of kifunensine on *N*-glycosylation modification and the production of rrBChE in a transgenic rice cell culture bioreactor. By adding kifunensine to the medium during bioreactor cultivation at the end of the growth phase and throughout the induction phase, we demonstrate the production and *N*-glycosylation pattern of rrBChE in culture medium and within the cell aggregates (cell-associated).

## 2. Results and Discussion

### 2.1. Cell Growth Kinetics, Sugar Consumption, and Oxygen Uptake Rate with Kifunensine Addition

**Figure 1:**
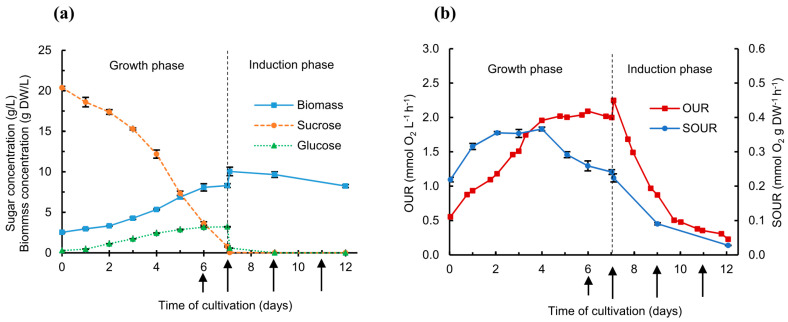
Transgenic rice cell culture grown in a 5 L bioreactor with the addition of 5 µM final concentration of kifunensine at day 6, 7, 9, and 11 of cultivation. (**a**) Growth profiles and sugar consumption, and (**b**) oxygen uptake rate (OUR) and specific oxygen uptake rate (SOUR). Error bars indicate the standard deviation from three biological replicates. Vertical dash lines indicate time of media exchange from sugar-rich medium to sugar-free medium. Arrows represent time of kifunensine addition to the rice cell suspension culture to achieve a final medium concentration of 5 µM, assuming 0 µM kifunensine prior to each addition.

The transgenic rice cell suspension culture was cultivated in a 5 L bioreactor with a working volume of 2.9 L. Figure 1a shows the growth and sugar concentration profiles during the cultivation period. With the initial biomass concentration (x_0_) of 2.53 ± 0.08 g DW/L, no lag phase was observed (data not shown). The maximum specific growth rate (µ_max_) was 0.21 ± 0.02 day^−1^, corresponding to a doubling time (τ_D_) of 3.3 ± 0.4 day (Table 1). The µ_max_ and τ_D_ in this study were consistent with our previous reports [39,43]. The media exchange from sugar-rich medium (NB+S) to 1.25X-concentrated sugar-free medium (NB-S) was performed at day 7 of cultivation when the final biomass concentration (x_f_) of the growth phase was 8.29 ± 0.24 g DW/L. As expected, the biomass concentration decreased from 10.04 ± 0.53 g DW/L (x’_0_) to 8.24 ± 0.19 g DW/L (x’_f_) during the induction phase (Figure 1a, days 7–12) due to sugar starvation. The reduction of biomass during sugar starvation was also noticed in our previous reports [39,43], suggesting that kifunensine may not adversely affect rice cell mass cultured in 1.25X NB-S, even though a negative impact on rice cell mass cultured in Chu medium without sucrose in the presence of kifunensine was previously reported [25].

Sucrose in the culture medium was hydrolyzed during heat sterilization (by autoclaving) and diluted by 20% *v/v* inoculation, leading to ~20 g/L at the beginning of cultivation (Figure 1a). The gradual decrease in sucrose concentration in the medium is caused by invertase enzymes in the rice cell wall that convert sucrose to glucose and fructose, which are then taken up by rice cells. At day 7 of cultivation, sucrose and glucose concentrations were 0.8 g/L and 3.2 g/L, respectively, before the media exchange, and 0 g/L and 0.6 g/L, respectively, after the media exchange. Even though sugar concentrations were not measured at day 8 of cultivation (day 1 following induction) in this study, our previous reports found that residual sucrose and glucose were usually depleted by the end of the first day of induction [39,42,43].

Figure 1b shows the profiles of oxygen uptake rate (OUR) (mmol O_2_ L^−1^ h^−1^) and specific oxygen uptake rate (SOUR) (mmol O_2_ g DW^−1^ h^−1^) over the course of the bioreactor run. During the growth phase, the OUR increased from day 0 to day 4 of cultivation, presumably due to exponential cell growth, and remained constant until the media exchange, suggesting the cells were in late exponential growth and the stationary phase between days 4 and 7. The maximum OUR during the growth phase was 2.09 ± 0.01 mmol O_2_ L^−1^ h^−1^ at day 6 of cultivation, while the maximum SOUR was 0.37 ± 0.01 mmol O_2_ g DW^−1^ h^−1^ at day 4 of cultivation (Table 1). The increase in OUR immediately after the media exchange was probably caused by the more concentrated biomass, allowing cells to consume the dissolved oxygen faster than the more diluted biomass prior to medium exchange. The OUR and SOUR decreased dramatically throughout the induction phase due to the lack of a carbon source, as also reported in previous studies [38,39,42,43], rather than the presence of kifunensine.

### 2.2. rrBChE Production and Recombinant Protein Purity in the Presence of Kifunensine

**Figure 2:**
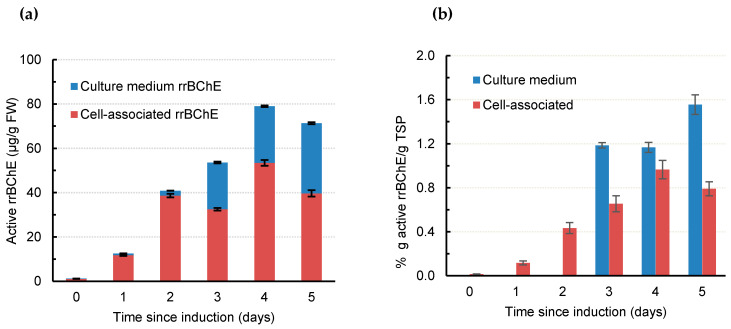
Production of rrBChE during the induction phase in a 5 L bioreactor. (**a**) Active rrBChE normalized by biomass fresh weight, and (**b**) rrBChE purity defined as % g active rrBChE per g total soluble protein (TSP). Error bars represent standard deviation from three technical replicates.

As shown in Figure 2a, the overall production level of active rrBChE increased by up to 80 µg/g FW, at least 1.5-fold higher than recently reported, using the same two-stage cultures [43]. Table 2 also shows a significant improvement in the volumetric productivity (680 ± 34 µg L^−1^ day^−1^) and specific productivity (82.5 ± 4.6 µg g DW^−1^ day^−1^). The increase in total active rrBChE, volumetric productivity, and specific productivity may be the result of increasing sugar-free medium (NB-S) concentration by 1.25 times and decreasing the bioreactor working volume during the media exchange by 1.25 times. Concentrated fresh NB-S might help to maintain the availability of micro- and macronutrients to ensure that cells have more precursors to produce more rrBChE, in the same way as adding concentrated amino acids enhances the production of recombinant human cytotoxic T-lymphocyte antigen 4-immunoglobulin (hCTLA4Ig) in fed-batch transgenic rice cell suspension cultures [44]. In addition, reducing the final working volume of the culture medium during media exchange increases the biomass concentration, with concentrates secreting rrBChE in the media. Choi et al. reported a decrease of acid α-glucosidase (GAA) concentration in transgenic rice cell culture medium treated with kifunensine only, but an increase in the presence of both kifunensine and swainsonine [25]. The discrepancy between our report and Choi et al.’s study may be due to different cell lines, nature and type of recombinant glycoproteins (GAA vs. BChE), culture vessels (bioreactor vs. flasks), physical parameters (agitation and aeration), method of kifunensine addition, and culture media components.

The amount of secreted active rrBChE in culture medium treated with kifunensine accounted for 44% of total active rrBChE at day 5 following induction compared with the negligible amount of culture medium rrBChE observed in our previous reports using similar bioreactor conditions [43]. In *Arabidopsis thaliana*, mutation of class I α-mannosidases (*mns*) genes resulted in swollen roots and altered cell wall structures in mutants [45]. The inhibition of class I α-mannosidases in the endoplasmic reticulum (ER) and Golgi by kifunensine may be thought to change rice cell wall structures and permeability, resulting in an enhancement of rrBChE excretion. Another possible explanation is that there might be a transporter in the secretory pathway of rice cells that explicitly recognize high-mannose rrBChE, leading to increased secreted rrBChE similar to mammalian cells. For example, the ER–Golgi intermediate compartment (ERGIC)-53, a transmembrane lectin localized in the ERGIC, specifically recognizes high-mannose glycoproteins and engages them into the secretory vesicles of the ERGIC for trafficking in mammalian cells [46]. Marcus and Perlmutter reported that the secretion of a mutant α1 antitrypsin Z in CJZ12B human fibroblast cell cultures was enhanced in the presence of kifunensine, probably via the ERGIC-53 cycling pathway [47]. However, investigation of the transporter(s) involved in the secretion of high mannose glycoproteins in rice cells is beyond the scope of the current study. In brief, kifunensine appears to enhance the excretion and/or the secretion of rrBChE in the culture medium.

The ratio of g active rrBChE to g total soluble protein (TSP), a measure that indicates rrBChE purity in crude extracts and/or the culture medium, is shown in Figure 2b. The active cell-associated rrBChE purity increased up to 0.97 ± 0.08% at day 4 following induction, which was slightly lower than that reported in our previous study, 1.41 ± 0.16% [43]. On the other hand, the maximum active culture-medium rrBChE purity was 1.55 ± 0.03% at day 5 following induction, implying that rrBChE in the culture medium was ~1.6 times purer than cell-associated rrBChE, while the amount of rrBChE in the culture medium was negligible under similar bioreactor conditions [43]. This could be interpreted as the presence of kifunensine enhances selective excretion and/or secretion of rrBChE. Although rrBChE can be purified from both biomass extracts and culture medium, a higher starting purity makes downstream processing easier. The higher purity of rrBChE in the medium confirmed that kifunensine improves the excretion and/or secretion of extracellular *N*-glycoproteins, since higher purity in cell-associated rrBChE is usually found in the absence of kifunensine [43].

### 2.3. Purification, SDS-PAGE, and Western Blot Analysis of rrBChE with/without Kifunensine Treatment

**Figure 3:**
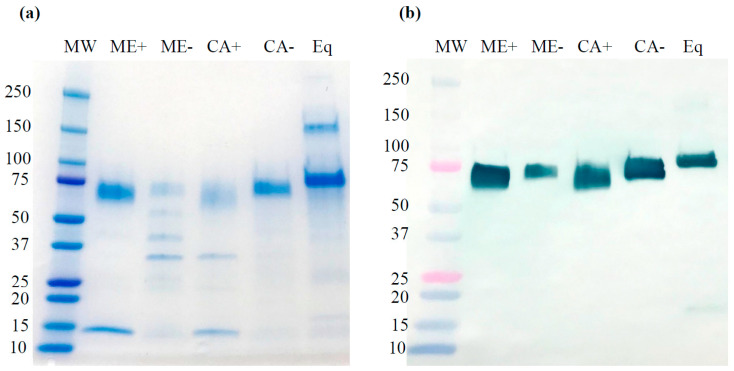
SDS-PAGE (**a**) and Western blot (**b**) run under reducing conditions for purified rrBChE with and without kifunensine treatment. Lane MW, molecular weight standard proteins; lane ME+ and lane ME-, 15 µL (~1580 ng rrBChE) and 30 µL (~70 ng rrBChE) of purified culture medium rrBChE with and without kifunensine treatment, respectively; lane CA+ and lane CA-, 30 µL (~585 ng rrBChE) and 15 µL (~1920 ng rrBChE) of purified cell-associated rrBChE with and without kifunensine, respectively; lane Eq, ~1680 ng of purified equine BChE. CA and ME stand for cell-associated rrBChE and culture medium rrBChE, respectively, while + and - refer to with and without kifunensine treatment, respectively.

Employing tangential flow filtration (TFF), DEAE anion exchange column chromatography, and Hupresin^®^ affinity chromatography, cell-associated rrBChE without kifunensine treatment was purified up to 94% purity (Figure 3a, lane CA-) through image analysis, consistent with our previous reports that used the same steps [2,3]. The impurities were likely host-cell proteins that nonspecifically bound to Hupresin^®^. Due to its small size, residual kifunensine was removed from the sample during the TFF and chromatography steps. However, culture medium rrBChE in the absence of kifunensine was only 31% purity (Figure 3a, lane ME-) likely due to a very low concentration of rrBChE (less than 3 µg/mL) in the culture medium at the beginning of purification. Even though TFF and DEAE were used for rrBChE enrichment, rrBChE purity was probably not high enough to prevent nonspecific binding of nontarget proteins to Hupresin^®^. Alkanaimsh et al. also found contaminating protein bands from Hupresin^®^ elution due to nonspecific binding when nonenriched *Nicotiana benthamiana* rBChE cell extract was loaded onto Hupresin^®^ [2]. Starting with low BChE purity, Onder et al. obtained 10–15% purity of hBChE purified from human blood plasma with a single-step Hupresin^®^, while 99% purity of hBChE was achieved when Q-ceramic ion exchange chromatography at pH 4.5 was used prior to the Hupresin^®^ step [48]. Thus, to acquire high rrBChE purity in the Hupresin^®^ elution, it may be advantageous to enrich rrBChE purity in the Hupresin^®^ loading solution.

For our samples generated in the presence of kifunensine, we started with low rrBChE concentrations in both cell extract and culture medium, causing low rrBChE purity in the Hupresin^®^ elution fractions (data not shown). However, the purity of rrBChE from cell extract and culture medium were increased to 67% and 76% (Figure 3a, lane CA+ and ME+), respectively, by buffer-exchanging the Hupresin^®^ combined elution and reloading onto Hupresin^®^ a second time. Repeating Hupresin^®^ chromatography purification significantly improves rrBChE purity when starting with a small amount of rrBChE, but this method is time consuming and cost-ineffective. Isolated rrBChE bands from each source, as shown in the Coomassie blue-stained SDS gel (Figure 3a), were excised and prepared for site-specific *N*-glycan analysis.

Figure 3b shows a Western blot of purified rrBChE from the medium and cell extract with and without kifunensine addition. The bands of cell extract rrBChE and culture medium rrBChE with and without kifunensine treatment were at 60–70 kDa and 65–75 kDa, respectively, consistent with previous studies performed in shake flasks (data not shown). This was likely due to the change in *N*-glycan structures in the presence of kifunensine. Furthermore, kifunensine also impacts cell extract and culture medium rrBChE *N*-glycoforms (described in Section 2.4), leading to the observed slightly different electrophoretic migration of the two rrBChE bands. Overall, SDS-PAGE and Western blot analysis identified purified rrBChE bands migrating differently, presumably due to different molecular weights associated with different *N*-glycan structures. The size of the rrBChE monomer was lower than the equine BChE monomer (which includes mammalian glycosylation), probably due to the differences between plant and mammalian *N*-glycan structures and/or site occupancy. Although the equine BChE site-specific glycosylation was not characterized, it was anticipated to be similar to hBChE. Only five *N*-glycosylation sites were detected in rrBChE in this study (Section 2.4), while seven *N*-glycosylation sites were occupied in hBChE [49], which could be responsible for the lower molecular weight of rrBChE compared to the equine BChE. Regardless of the different site occupancies and *N*-glycan structures, our previous study demonstrated comparable enzyme kinetic parameters, such as K_m_ and k_cat_ in 0.1 M sodium phosphate buffer of pH 7.4 at 25 ˚C, compared to hBChE under the same conditions [3].

### 2.4. Site-Specific N-glycosylation Analysis

**Figure 4:**
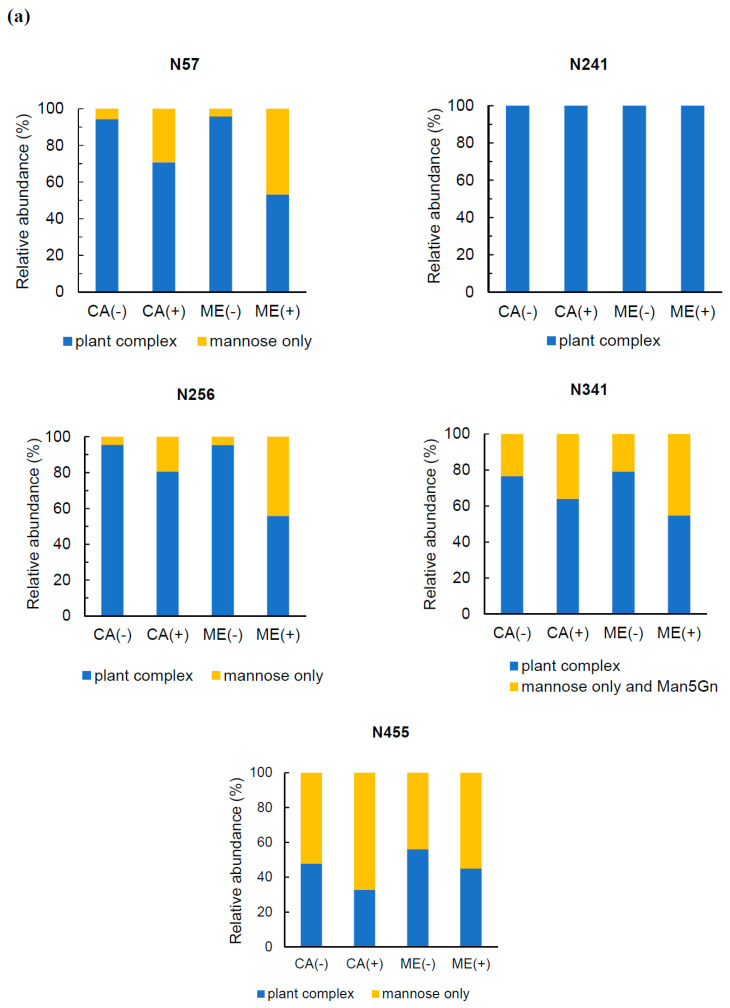

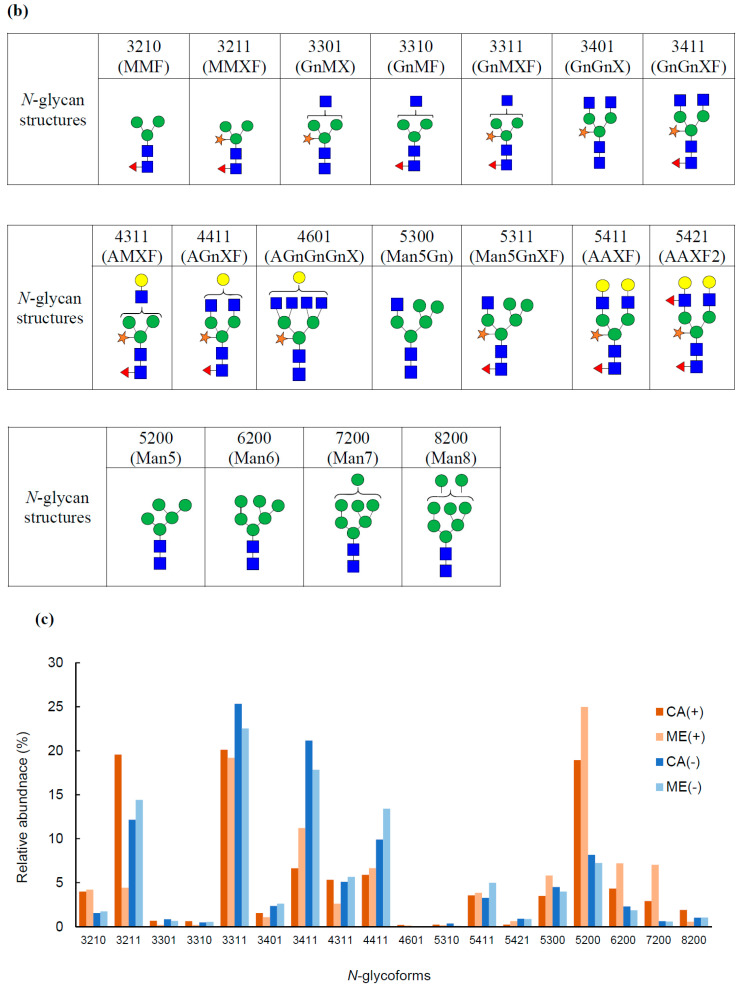
Site-specific *N*-glycan analysis of purified cell-associated rrBChE and culture medium rrBChE with and without kifunensine treatment. (**a**) The relative abundance (%) of plant-complex glycoforms (all glycoforms except for 5200, 5300, 6200, 7200, and 8200) and mannose-only (5200, 6200, 7200, and 8200) or mannose-only and Man5Gn (5200, 5300, 6200, 7200, and 8200) glycoforms of each site; CA(-) and CA(+), cell-associated rrBCHE with and without kifunensine treatment, respectively; ME(-) and ME(+), culture medium rrBChE with and without kifunensine treatment, respectively. (**b**) Structures of *N*-glycans found in rrBChE; blue square (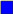), *N*-acetylglucosamine; green circle (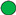), mannose; yellow circle (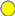), galactose; red triangle (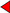), fucose; orange star (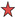), xylose. (**c**) The relative abundance (%) of overall (combining all sites) *N*-glycoform distributions of rrBChE with and without kifunensine treatment. CA and ME stand for cell-associated rrBChE and culture medium rrBChE, respectively, while + and - refer to with and without kifunensine treatment, respectively.

The *N*-glycan structures of purified cell-associated and culture medium rrBChE with and without kifunensine treatment were mapped by LC-MS/MS and quantified by triple quadrupole (QQQ). The number given to each *N*-glycan indicated the number of hexoses, *N*-acetylhexoseamine, fucose, and xylose, respectively. Figure 4a reveals five *N*-glycosylation sites (N57, N241, N256, N341, and N455) of rrBChE that were observed, quantified, and normalized per each site in this study, while Figure 4b shows the structure of *N*-glycans. Although site N481 was previously reported in cell-associated rrBChE without kifunensine treatment [3], this site was not observed in this study, perhaps due to the different sample preparation procedures and low abundance of the glycopeptides. Overall, in the presence of kifunensine, the relative abundance of the plant-complex glycoforms of rrBChE substantially decreased, while the relative abundance of mannose-only (mannose-only and Man5Gn, since it does not include plant-specific glycoforms) glycoforms noticeably increased at all *N*-glycosylation sites, except site N241 (Figure 4a). Plant-specific sugars, namely, β-1,2-xylose and core α-1,3-fucose, in complex *N*-glycans such as MMXF (3211), GnMXF (3311), and GnGnXF (4411), were primarily found in rrBChE without kifunensine treatment, as expected (Figure 4c and Figure S1). Figure 4c shows that the relative abundance of Man5/6/7/8 structure significantly increased in both cell-associated and culture medium rrBChE treated with kifunensine, even though some complex *N*-glycans were still observed, suggesting incomplete inhibition of α-mannosidase I from mannose trimming. In addition, regardless of the presence of kifunensine, as seen in Figure 4c, the relative abundance of GnMXF (3311) appeared to be conserved between cell-associated and culture medium rrBChE for an unknown reason.

*N*-glycoforms of rrBChE from cell extract and culture medium in the absence of kifunensine are comparable in all *N*-glycosylation sites (Figure 4c and Figure S1), indicating that rrBChE from the culture medium was fully glycosylated throughout the secretory pathway and secreted outside the cell membrane. It could also be interpreted that the majority of cell-associated rrBChE analyzed at day 5 following induction was secreted outside of the cell membrane but likely trapped in apoplast, cell wall matrix, and/or cell aggregates. In contrast, culture medium and cell-associated rrBChE produced under kifunensine treatment showed significantly different *N*-glycoforms, implying interference in the *N*-glycosylation pathway due to partial α -mannosidase I inhibition by kifunensine. For example, in the presence of kifunensine, the relative abundances of rrBChE Man5/6/7 structures from the culture medium, ME(+) and from the cell extract, CA(+) were 47% and 29% at site N57, respectively, and 44% and 19% at site N256, respectively (Figure 4a and Figure S1), indicating further mannose trimming of intracellular rrBChE by functional α-mannosidase I. Perhaps it takes some time for kifunensine to be taken up by the rice cells and transported to the ER and the Golgi to inhibit α-mannosidases I, and in the meantime, some rrBChE molecules are glycosylated in a traditional manner because α-mannosidases I are functional.

Interestingly, the most abundant *N*-glycan structure at site N241 was 3211 in CA(-), CA(+), and ME(-), but 4601 in ME(+) (Figure S1). Per molecular dynamic simulations, the presence of the *N*-glycan at site 241 plays a key role in catalytic activity and interacts with the BChE tetramerization domain [50]. The reason for the *N*-glycoform shift from predominantly 3211 to 4601 at site N241 in ME(+) (Figure S1) is unclear; however, it does not imply that rrBChE gained or lost activity and/or interaction with the tetramerization domain, since the N241 site remained highly glycosylated. At site N341, the abundance of the Man5/6 structure increased in the presence of kifunensine, while the abundance of 5300 increased in ME(+) only. The Man7/8 structure was the main *N*-glycoform found in CA(+) and ME(+) at site N455, whereas CA(-) and ME(-) demonstrated more complex *N*-glycoforms than CA(+) and ME(+) at this site. The multitude of *N*-glycosylation sites in rrBChE may be why incomplete inhibition of α-mannosidase I was observed, leading to further mannose trimming and oligosaccharide processing in the ER and/or Golgi even in the presence of 5 µM kifunensine in the bioreactor culture. Nonetheless, overall kifunensine mass-transfer limitations from the culture medium to cell aggregates, through the cell wall matrix, cell membrane, and into the cis-Golgi and ER, where α-mannosidase I is located, was probably the major reason for incomplete inhibition of α-mannosidase I, leading to the Man5/6/7/8 structure being found in rrBChE treated with kifunensine rather than the expected Man9 structure.

In plant-based systems, several studies reported that glycoproteins treated with kifunensine contained predominantly Man9 structures [4,23,24] and Man7/8/9 structures [25], while Man5/6/7 was the main structure found in this study. This discrepancy is likely due to the mass-transfer limitation of kifunensine. Our hypothesis is that the rate of mannose trimming by ER and/or Golgi α-mannosidase I is faster than the rate of diffusion of kifunensine from cell aggregates to individual cells and from individual cells to Golgi and ER compartments. Perhaps the time-scale for kifunensine transport from bulk liquid to cell aggregate surface, diffusion in the aggregate pores, transport through the cell wall matrix, transport across the cell membrane and transport across the Golgi membrane and/or the ER membrane, is longer than 24 h, therefore the concentration at α-mannosidase I sites is not high enough for complete inhibition at the start of the rrBChE production phase. It is known that plant cells tend to grow into large aggregates; for example, our group previously reported that the mean aggregate size was around 500 µm for rice cell suspension culture in AA medium [51]. Additionally, in this study we observed an average size of rice cell aggregates primarily larger than 1500 µm in NB medium (data not shown), which may have led to the kifunensine diffusion limitation. The transgenic rice cell suspension cultures grown in Chu medium in 100 mL shake flasks for acid α-glucosidase (GAA) production studied by Choi et al. may have been of smaller average aggregate size and carrying less kifunensine diffusion limitation compared to ours in the presence of 5 µM of kifunensine [25].

On the other hand, in the whole-plant *N. benthamiana* system, vacuum agroinfiltration of *A. tumefaciens* suspension containing 0.25–5 µM kifunensine [23] or 5.4 µM kifunensine [4] and hydroponic kifunensine (5 µM) treatment after vacuum agroinfiltration [24] could enhance kifunensine transport by directly introducing the kifunensine solution to intercellular spaces. Moreover, kifunensine transport into plant cytoplasm may be enriched at the wounded sites of plant cells infected by *A. tumefaciens,* resulting in strong inhibition of α-mannosidase I. For instance, the relative abundances of Man9 structure in target glycoproteins were 87% [23], 42% [4], and 64.5% [24] when treated with kifunensine of 0.375 µM, 5.4 µM, and 5 µM, respectively. Different target glycoproteins with different numbers of *N*-glycosylation sites may be another reason for the observed differences in relative abundance of Man9 structures between the three studies. In addition, Kommineni et al. reported a relative abundance of 45% hybrid glycoforms (3411, 3401, and 3400) and 55% Man 6/7/8/9 structures in the glycoprotein (rituximab) treated with 0.25 µM kifunensine in agroinfiltration solution [23], indicating incomplete inhibition of α-mannosidase I, likely due to the limited kifunensine concentration inside the cellular ER and/or Golgi compartments. This suggests that higher kifunensine concentration could improve oligomannose glycans and reduce plant-specific *N*-glycans in plant cell suspension cultures, especially in cell lines that grow in large aggregates.

Recombinant BChE (rBChE) from the milk of transgenic goats demonstrated different glycan structures compared to hBChE, especially in terms of sialic acid content, but the differences may not impact its biological activity [52], suggesting that plant-cell derived BChE, such as rrBChE, probably possesses comparable biological activity to mammalian-derived BChE, regardless of glycosylation pattern. For example, the inhibition rate constants (k_i_) of five organophosphates (OP), tabun, sarin, soman, cyclosarin, and methylphosphonothioic acid, for rrBChE and hBChE exceeded the minimum values needed to protect against OP intoxication in an in vitro study [3]. Although the concentration of rrBChE of 7.5 mg/L in the transgenic rice cell suspension culture in this study was significantly lower than the concentration of recombinant BChE of 1–5 g/L from the milk of transgenic goats [52], it is important to consider the long development time required between gene transfer and lactation for transgenic goats. There is limited quantitative information on production costs for rBChE from the milk of transgenic goats. However, since mammalian production systems can harbor and propagate human pathogens, regulatory requirements for the characterization of the transgenic founder, as well as feeding, housing, health monitoring, genetic stability assessment, and regulated disposal of ex-producer animals, could increase the production costs of human therapeutics in transgenic animals compared with plant-based systems.

## 3. Materials and Methods

### 3.1. Transgenic Rice Cell Suspension Inoculum

The expression vector design, cloning, transformation and selection of the callus, and media components were previously described [39]. In brief, rice calli derived from *Oryza sativa* cv. Taipei 309 embryo/scutellum were co-cultured with *Agrobacterium tumefaciens* containing the binary vector with the RAmy3D promoter, codon-optimized human BChE gene to express in rice, the RAmy3D signal peptide, and the RAmy3D terminator. After eight rounds of screening starting with more than 300 transformation events, a stable transgenic rice cell line “9-2” was established [39], which was previously used in other studies [3,39,43] as well as this study. The inoculum cultures were grown in 250 mL sugar-rich media (modified Chu/Gamborg medium, NB + S, as previously described [39,43]) in 1 L-shake flasks for 6 days in an Innova 4000 incubator/shaker (Eppendorf, Inc., Hauppauge, NY, USA) at 140 rpm and 27 °C in the dark.

### 3.2. Bioreactor Operation with Kifunensine Treatment

Combined shake flasks described in Section 3.1 were inoculated at about 20% *v/v* (volume of inoculating suspension to final working volume of culture) in a 5 L stirred-tank bioreactor (BioFlo 3000, formerly New Brunswick Scientific, Eppendorf, Inc., Huappauge, NY, USA) equipped with a pitched blade impeller (10.2 cm diameter) and containing 2.5 L of sterile NB + S added through the headplate inoculation port inside a biosafety cabinet. Bioreactor conditions were controlled at 27 °C, 75 rpm agitation speed, 40% dissolved oxygen (DO) of air saturation (O_2_ sensor, Mettler Toledo, Billerica, MA, USA), and 0.2 vvm (volume of gas sparged per bioreactor working volume per minute) of the overall mixed gas (compressed air, N_2_ and O_2_) flow rate. The oxygen uptake rate (OUR) was measured by the change in DO level in the culture when aeration is stopped but with continued agitation. The culture pH (pH sensor, Mettler Toledo, Billerica, MA, USA) was monitored but not controlled. The cultures grown in the glass bioreactor were exposed to ambient light.

Freshly prepared kifunensine (Cayman Chemical, Ann Arbor, MI, USA) solution was added to the bioreactor at 5 µM final concentration in the bioreactor medium at day 6 of cultivation, 24 h before the media exchange. At day 7 of cultivation, media exchange was performed to replace spent sugar-rich medium (NB+S) with sugar-free medium (NB-S) using the same method as previously described [39]. Kifunensine solution was added at day 0 (right after the media exchange), day 2, and day 4 following induction, assuming 0 µM of kifunensine in the bulk medium prior to each kifunensine addition. During the cultivation, four independent samples were taken at a given time point for fresh and dry weight analysis, sugar analysis, and quantification of rrBChE and total soluble protein (TSP).

### 3.3. Harvesting of Culture Medium rrBChE and Cell-Associated rrBChE

The bioreactor run was terminated at day 5 following induction (day 12 of cultivation) and the rice cell aggregates were allowed time to sediment (~10 min). The culture medium was withdrawn from the bioreactor through a sampling port using a peristaltic pump and stored at 4 °C The bioreactor headplate was then opened. Rice cell biomass was collected and vacuum-filtered on Whatman Grade 1 (Cytiva, formerly GE Healthcare Life Sciences, Marlborough, MA, USA). Fresh biomass was weighed and stored at −20 °C.

### 3.4. Biomass Measurements

The method for biomass measurement was described earlier [39]. In brief, the total culture volumes for three samples (~10 mL each) taken from the bioreactor were recorded and then centrifuged at 3750 rpm (3200× *g*; Beckman GS-6KR, Beckman Coulter, Inc., Brea, CA, USA). Then, the supernatant was removed and stored at 4 °C for sugar analysis and protein quantification. The biomass was washed with double-distilled water, vacuum-filtered onto a 1.6 µm Binder-Free Glass Microfiber filter (Whatman GF/A 4.7 cm; Cytiva, formerly GE Healthcare Life Sciences, Marlborough, MA, USA), weighed for fresh biomass, dried in an oven at 65 °C for 24 h, and weighed until the weight no longer changed to determine the dry biomass.

### 3.5. Sugar Analysis

A YSI 2900 Biochemistry Analyzer (Xylem, Inc., Rye Brook, NY, USA) was used to quantify the concentrations of sucrose and glucose in the culture medium (supernatant from Section 3.4). The samples were diluted with double-distilled water, if necessary, to ensure that the sugar concentrations fell into the calibration range.

### 3.6. Active rrBChE and Total Protein Quantification

To quantify active rrBChE and TSP associated with rice cells, extraction buffer (100 mM sodium phosphate buffer, 100 mM NaCl, pH 7.4) was added to cell mass at a 1 mL buffer per 1 g FW ratio (1:1 *v/w*). The mixture was homogenized with a Tissue Tearor (Biospec Products, Bartlesville, OK, USA) for 30 s and centrifuged at 14,000 rpm (20,817× *g*; Eppendorf 5417R, Eppendorf, Inc., Hauppauge, NY, USA) for 5 min at 4 °C. The supernatant was collected and stored at 4 °C for further analysis. In addition, the supernatant collected as described in Section 3.4 was used to quantify rrBChE and TSP in the culture medium. The concentration of active rrBChE was determined using a modified Ellman activity assay [53] as previously described [39], assuming a specific activity of 260 U/mg for crude rrBChE. The assay monitored the hydrolysis reaction of S-butyrylthiocholine (Sigma-Aldrich, St. Louis, MO, USA) by active rrBChE in the presence of 5,5′-dithiobis-2-nitrobenzene (Sigma-Aldrich, St. Louis, MO, USA) in 0.1 M sodium phosphate buffer, pH 7.4. The reaction was performed in triplicate, and the absorbance was monitored at 405 nm for 3 min at 25 °C using a spectrophotometer (SpectraMax 340PC, Molecular Devices, Sunnyvale, CA, USA). Samples were sometimes diluted to obtain the rate of increasing absorbance at 200–500 mOD/min. Total soluble protein (TSP) was measured using the Bio-Rad Protein Assay kit (Bio-Rad, Hercules, CA, USA), following the manufacturer’s instructions.

### 3.7. Purification of rrBChE

For cell-associated rrBChE purification, several steps were employed, including rice cell homogenization/extraction, centrifugation, microfiltration, tangential flow filtration (TFF) for ultrafiltration (UF) and diafiltration (DF), anion exchange column chromatography using DEAE FF HiScreen prepacked column (Cytiva, formerly GE Healthcare Life Sciences, Marlborough, MA, USA), and Hupresin^®^ (CHEMFORASE, Rouen, France) affinity chromatography, as previously described [3]. The extraction buffer, diafiltration buffer, and DEAE equilibration and washing buffer were 20 mM sodium phosphate buffer, pH 7.4, while the DEAE elution buffer was 20 mM sodium phosphate buffer, 300 mM NaCl, pH 7.4. For Hupresin^®^ chromatography, the equilibration and washing buffer was 20 mM sodium phosphate buffer, 150 mM NaCl, pH 7.4, whereas the elution buffer was 20 mM sodium phosphate buffer, 150 mM NaCl, 500 mM tetramethylammonium chloride (TMAC), pH 7.4. All chromatography flow rates were 1 mL/min with isocratic elution over five column volumes.

Purification of rrBChE from the culture medium started with centrifugation to remove suspended particles prior to microfiltration. Then, TFF, DEAE anion exchange chromatography, and Hupresin^®^ chromatography were applied using the same buffers and flow rates as mentioned above. All elution fractions were stored at 4 °C for further analysis, such as modified Ellman assay, Bradford assay, SDS-PAGE, and Western blotting.

### 3.8. Gel Electrophoresis and Western Blot

Samples for SDS-PAGE were prepared and run for 37 min at 200 V under reducing and denaturing conditions using precast 4–20% Tris-glycine gradient gels (Bio-Rad, Hercules, CA, USA). Equine BChE (SigmaAldrich, St. Louis, MO, USA) was used as a reference standard. After electrophoresis, Coomassie Brilliant Blue G-250 (Bio-Rad, Hercules, CA, USA) was used to stain the gel to quantify rrBChE purity using ImageJ software (version 1.53a, US National Institutes of Health, Bethesda, MD, USA), with a second gel transferred to a nitrocellulose membrane at 100 V for 80 min using Tris/Glycine/Methanol as a transfer buffer. After blotting, the nitrocellulose membrane was blocked overnight at 4 °C with 5% nonfat dried milk (NFDM) in phosphate buffer (PBS) to prevent nonspecific binding. A mouse anti-BChE IgG (1:200) (Santa Cruz Biotechnology, Santa Cruz, CA, USA) as a primary antibody and a goat anti-mouse IgG-HRP as a secondary antibody (1:2000) (Santa Cruz Biotechnology, Santa Cruz, CA, USA) were used. Then, 3,3′,5,5′-tetramethylbenzidine (TMB) substrate (Promega, Madison, WI, USA) was used to develop immunoreactive bands.

### 3.9. Site-Specific N-glycopeptide Analysis of rrBChE

The Coomassie-stained target protein gel bands from 3.8 were excised and cut into small pieces. Gel pieces were then treated with 100 mM ammonium bicarbonate (NH_4_HCO_3_) and 100% acetonitrile (ACN) alternatively for four cycles and dried under vacuum completely. For the *N*-glycopeptide analysis, gel pieces were suspended in 100 mM NH_4_HCO_3_ solution and digested with 2 µg of trypsin for 18 h in a 37 °C water bath. The supernatant containing digested proteins was collected, followed by the extraction of trapped peptides by sonicating gel pieces with a solution of 5% formic acid and 60% ACN. The combined solution was dried in a vacuum prior to mass spectrometry (MS) analysis.

The sample was reconstituted with Milli-Q water and 5 µL of the sample was subjected to MS analysis. An LC-MS system with an Agilent 1290 infinity ultra-high-pressure liquid chromatography (UHPLC) system (Agilent Technologies, Inc., Santa Clara, CA, USA) coupled to an Agilent 6495 triple quadrupole (QQQ) mass spectrometer (Agilent Technologies, Inc., Santa Clara, CA, USA) was employed for the analysis. For the separation of peptides and glycopeptides, an Agilent Eclipse plus C18 column (RRHD 1.8 µm, 2.1 × 150 mm) was used with an Agilent Eclipse plus C18 guard column (RRHD 1.8 µm, 2.1 × 5 mm) as a guard column. To conduct the targeted glycopeptide quantitation, a transition list of targeted glycopeptides was applied for the dynamic multiple reaction monitoring (dMRM) mode of the mass spectrometer. Precursor ions of the targeted glycopeptides were selected and fragmented, with several common oxonium fragments, with m/z values such as 204.08 and 366.14, detected as product ions. For the quantitation of glycopeptides, Agilent MassHunter Quantitative Analysis software (version B.05.02, Agilent Technologies, Inc., Santa Clara, CA, USA) was used.

## 4. Conclusions and Future Prospects

Performing a media exchange using 1.25-times-concentrated sugar-free medium (NB-S) together with 1.25-times-reduced culture volume and addition of kifunensine prior to and after the media exchange resulted in increased total production levels of active rrBChE, volumetric productivity, and specific productivity by 1.5 times, 3.4 times, and 1.5 times, respectively, compared with a bioreactor run with same operating conditions (27 °C, 75 rpm, 0.2 vvm) but no kifunensine treatment. Moreover, kifunensine enhanced the excretion of recombinant rrBChE glycoprotein through the secretory pathway, leading to 44% of total rrBChE in the culture medium at day 5 following induction and increasing extracellular rrBChE purity to 1.6% rrBChE/TSP compared with 0.8% cell-associated rrBChE/TSP. Coomassie-stained SDS-PAGE and Western blot analyses showed different migration bands of rrBChE with and without kifunensine treatment due to different *N*-glycan structures. *N*-Glycosylation site-specific analysis revealed increased oligomannose glycans at site N57, N246, N341, and N455 in both purified cell-associated and culture medium-derived rrBChE in the presence of kifunensine, while the mass transfer limitation of kifunensine was thought to be the main reason for the weak inhibition of α-mannosidase I in this bioreactor study.

At the laboratory scale, we produced ~16 mg of rrBChE in a 2 L working volume during a 12-day batch run, corresponding to a volumetric productivity of 0.680 mg L^−1^ day^−1^. A technoeconomic model developed for semicontinuous large-scale production (25 kg/year) of rrBChE (without kifunensine addition) at a higher volumetric productivity (1.5 mg L^−1^ day^−1^) showed that the process could be cost-effective with a cost-of-goods sold of ~$660/gram, less than 3% of the estimated cost of plasma-derived hBChE at ~$25,000/gram [54].

The addition of compounds to the culture medium to alter the function of glycan-modifying enzymes is the simplest method to modify *N*-glycan structure of a target glycoprotein compared to other methods. As a bioprocessing approach, it does not require alteration of the primary amino acid sequence of the target protein (i.e., various product glycoforms can be produced from the same transgenic cell line), or time-consuming glycoengineering of the host that could impact cell growth or viability, yet still allows secretion of the product into the culture medium. For example, adding kifunensine for *N*-glycan modification is a simple and effective way of obtaining oligomannose glycoproteins with reduced plant-specific xylose and fucose moieties. However, this method may not be cost-effective in large-scale production depending on the production level and market price of the glycoprotein product, the amount and frequency of kifunensine addition (which is not currently optimized and is likely to be cell line- and product-specific), and the price of kifunensine in bulk quantities. Currently, at our laboratory-scale pricing for kifunensine ($23/mg, Cayman Chemical, Ann Arbor, MI, USA), the addition of 5 µM kifunensine in NB-S increases induction medium costs by ~14-fold and contributes ~$225 in reagent costs for the 5 L bioreactor run. Although the cost of the growth and induction medium (even including kifunensine) is still significantly lower than mammalian cell culture medium, and the price of kifunensine is likely to decrease more than 10-fold with larger demand and bulk pricing, it may be advantageous to reduce bioreactor working volume during the induction phase to minimize kifunensine cost, enhance mass transfer, and concentrate extracellular rrBChE. Employing current genomic editing techniques such as CRISPR/Cas9 to knock out *XylT* and *FucT* genes to remove plant-specific α-1,3 fucose and β-1,2 xylose in host rice lines, similar to what was done in *N. tabacum* BY-2 cell lines without negative impacts in terms of cell growth rate [10,11], would be worth investigating as an alternative to modify *N*-glycans of secreted glycoproteins, such as rrBChE, for large-scale operations.

## Figures and Tables

**Table 1:** Growth parameters of transgenic rice cells grown in a 5 L bioreactor. Errors in biomass concentrations indicate one SD from three biological replicates, while others indicate one SD from regression analysis.

t_growth_ (Days)	x_0_ (g DW/L)	x_f_ (g DW/L)	µ_max_ (Day^−1^)	τ_D_ (Days)	Max OUR (mmol O_2_ L^−1^ h^−1^)	Max SOUR (mmol O_2_ g DW^−1^ h^−1^)
7	2.53 ± 0.08	8.29 ± 0.24	0.21 ± 0.02	3.3 ± 0.4	2.09 ± 0.01	0.37 ± 0.01

Note: t_growth_ indicates cultivation time during the growth phase, x_0_ and x_f_ are the initial and final biomass concentrations in the growth phase, respectively, µ_max_ is the maximum specific growth rate, τ_D_ is the doubling time, OUR is the oxygen uptake rate, and SOUR is the specific oxygen uptake rate.

**Table 2:** Rice recombinant BChE (rrBChE) production parameters during the induction phase. Errors in biomass concentrations and maximum total rrBChE production indicate one SD from three biological replicates and one SD from three technical replicates, respectively. Errors in maximum volumetric productivity and maximum specific productivity represent one SD from error propagation.

t_induction_ (Days)	x’_0_ (g DW/L)	x’_f_ (g DW/L)	g FW/ g DW Ratio†	Max total rrBChE Production (µg/g FW; mg/L)	Max Volumetric Productivity † (µg L^−1^ Day^−1^)	Max Specific Productivity † (μg g DW^−1^ Day^−1^)
5	10.04 ± 0.53	8.24 ± 0.19	11.5 ± 0.3	79.0 ± 2.2; 7.5 ± 0.4	680 ± 34	82.5 ± 4.6

Note: t_induction_ indicates cultivation time during the induction phase, x’_0_ and x’_f_ are the initial and final biomass concentrations in the induction phase, respectively, and **†** indicates that the fresh weight (FW) to dry weight (DW) ratio at day 12 of cultivation was used to determine maximum volumetric productivity and maximum specific productivity at day 11 of cultivation due to a lack of FW and DW data at day 11.

## Supplementary Materials

Supplementary materials can be found at https://www.mdpi.com/1422-0067/21/18/6896/s1.
