# Supplementary material for: Effects of Kifunensine on Production and N-Glycosylation Modification of Butyrylcholinesterase in a Transgenic Rice Cell Culture Bioreactor
Source: Int J Mol Sci. 2020 Sep 20;21(18):6896. doi: 10.3390/ijms21186896 (PMC7555773; doi:10.3390/ijms21186896)
Supplement: Supplementary file 1 [file ijms-21-06896-s001.pdf]

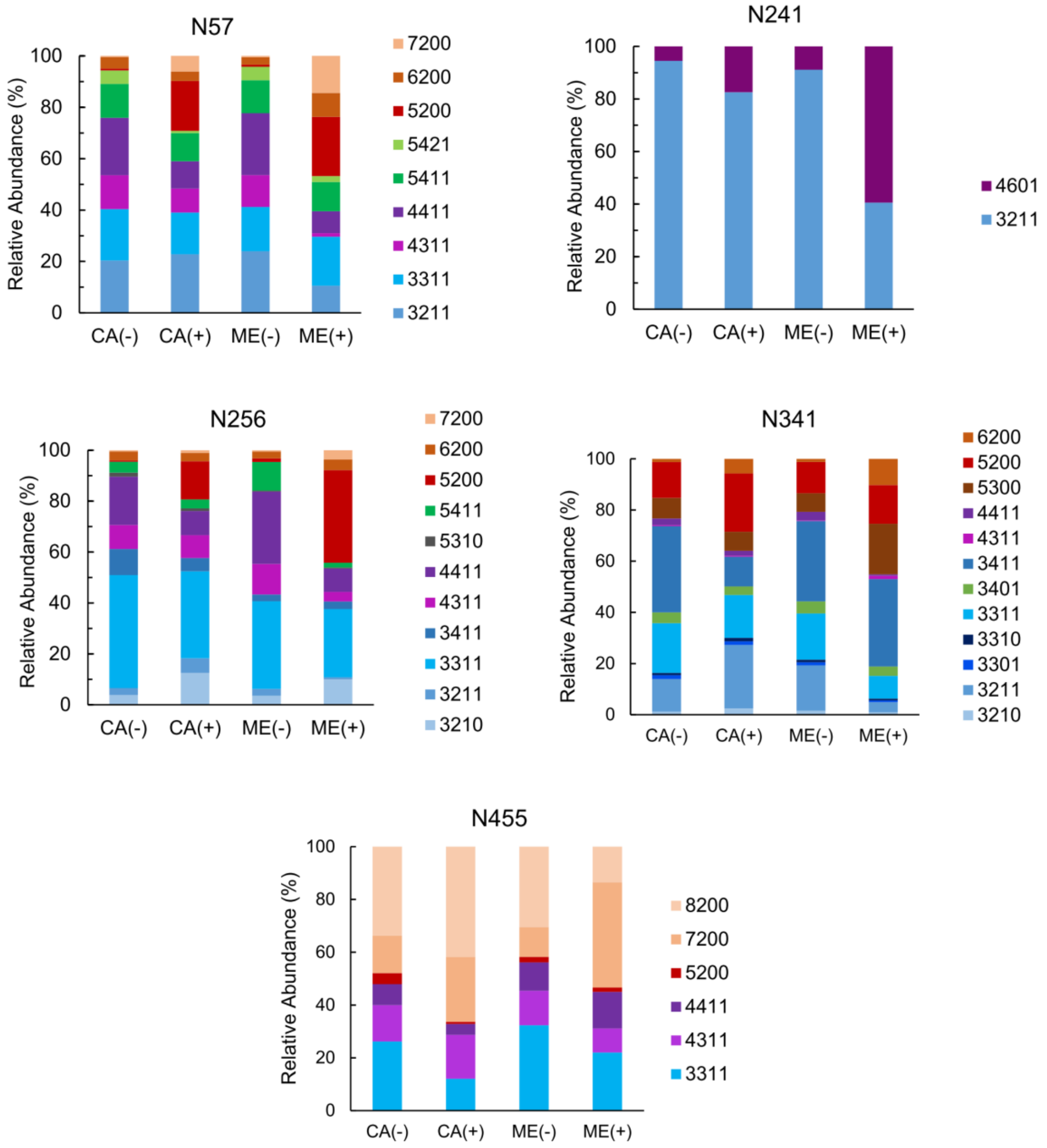

Figure S1. Site-specific N-glycan analysis of purified cell-associated rrBChE and culture medium rrBChE with and without kifunensine treatment. Glycoform distribution of each site analyzed by LC-MS/MS representing in % relative abundance; CA(-) and CA(+), cell-associated rrBCHE with/without kifunensine treatment, respectively; ME(-) and ME(+), culture medium rrBChE with/without kifunensine treatment, respectively. CA and ME stand for cell-associated rrBChE and culture medium rrBChE, respectively, while + and – refer to with and without kifunensine treatment, respectively.
